# Liquid Crystal Elastomers for Biological Applications

**DOI:** 10.3390/nano11030813

**Published:** 2021-03-22

**Authors:** Mariam Hussain, Ethan I. L. Jull, Richard J. Mandle, Thomas Raistrick, Peter J. Hine, Helen F. Gleeson

**Affiliations:** School of Physics and Astronomy, University of Leeds, Leeds LS2 9JT, UK; phymhu@leeds.ac.uk (M.H.); e.i.l.jull@leeds.ac.uk (E.I.L.J.); r.mandle@leeds.ac.uk (R.J.M.); py14tr@leeds.ac.uk (T.R.); p.j.hine@leeds.ac.uk (P.J.H.)

**Keywords:** liquid crystal elastomers, biological materials, auxetics, biomimetics, actuators

## Abstract

The term liquid crystal elastomer (LCE) describes a class of materials that combine the elastic entropy behaviour associated with conventional elastomers with the stimuli responsive properties of anisotropic liquid crystals. LCEs consequently exhibit attributes of both elastomers and liquid crystals, but additionally have unique properties not found in either. Recent developments in LCE synthesis, as well as the understanding of the behaviour of liquid crystal elastomers—namely their mechanical, optical and responsive properties—is of significant relevance to biology and biomedicine. LCEs are abundant in nature, highlighting the potential use of LCEs in biomimetics. Their exceptional tensile properties and biocompatibility have led to research exploring their applications in artificial tissue, biological sensors and cell scaffolds by exploiting their actuation and shock absorption properties. There has also been significant recent interest in using LCEs as a model for morphogenesis. This review provides an overview of some aspects of LCEs which are of relevance in different branches of biology and biomedicine, as well as discussing how recent LCE advances could impact future applications.

## 1. Introduction

Liquid crystal elastomers (LCEs) are a novel class of materials that combine the properties of liquid crystals (which exhibit orientational order) with the elastic properties of conventional elastomers [1]. As a result, they display unique and interesting responses to a variety of external stimuli, as well as intriguing responses to mechanical deformation, such as semi-soft elasticity, auxeticity and actuation properties. The exceptional potential of these elastomers is constantly being expanded on as more advanced synthesis and characterisation techniques are being developed. An interesting example is recent tensile rig developments which have allowed the concurrent analysis of the tensile behaviour and the liquid crystal texture. The resulting insight into the strain-dependent liquid crystal director reorientation, the mechanical properties and the birefringence (and hence the liquid crystal order parameter), led to a re-evaluation of deformation modes that occur in different LCE systems [2]. The exceptional properties of LCEs, such as stimuli responsiveness and actuation, have shown them to be versatile materials for use in a range of applications in the fields of biology and medicine, from artificial muscles to the control of cell maturation during cell culture [3]. This review article will cover some of the key discoveries and advances of LCEs in the field of biology.

## 2. Background to Liquid Crystal Elastomers and Liquid Crystals

A liquid crystal is a thermodynamically stable phase characterised by the anisotropy of properties without the existence of a three-dimensional crystal lattice, existing in the temperature window between the solid (crystalline) and isotropic (liquid) phase; therefore, liquid crystal phases are referred to as mesophases [4]. If these phases are formed as a result of a temperature change, they are referred to as “thermotropic” phases. These mesophases can also form as a result of amphiphilic materials dispersed in an appropriate solvent—these are “lyotropic” liquid crystal phases [5].

**Figure 1:**
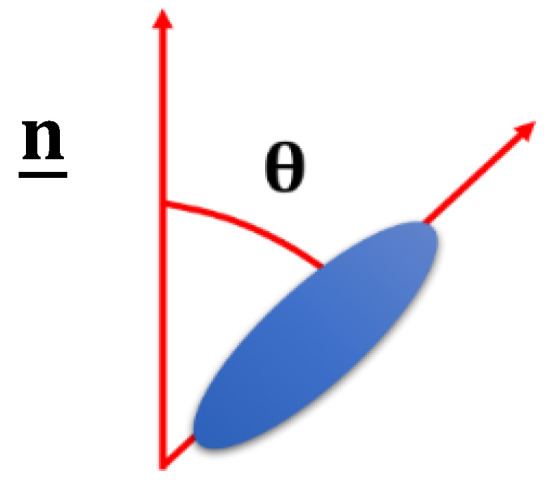
Schematic showing the angle *θ* between the director, $\underline{n}$ and the long axis of a molecule.

**Figure 2:**
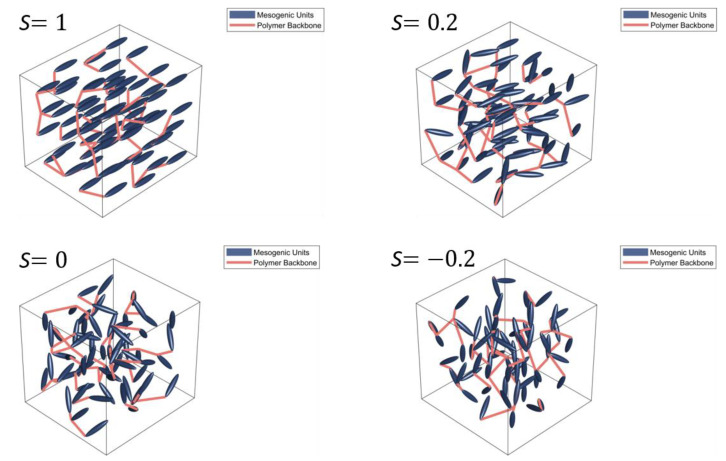
Schematic displaying the order of a liquid crystal polymer network. It clearly displays the impact of the order on the polymer backbone shape and orientation. Presented here are the results for a *S* value of *S* = 1, *S* = 0.6, *S* = 0, and *S* = −0.2. The director, $\underline{n}$, is parallel to the x axis.

Liquid crystalline materials usually have a few distinctive characteristics, including: molecular shape anisotropy, a strong dipole and/or easily polarisable substituents. Further, the liquid crystal molecules (mesogens) tend to point along a common axis, referred to as the director. In contrast, liquids have no preferred order, whereas the molecules in solids are highly ordered with very limited translational freedom. The orientational order of a liquid crystal mesophase lies between that of a liquid (which is zero) and that of a crystalline solid (which is 1) [6]. This orientational order can be defined by the order parameter, *S*, as follows:

$$S=\;\langle P_{2}\cos\left(\theta )\right\rangle =\frac{1}{2}\left\langle (3\;\cos^{2}\left(\theta \right)-1)\right\rangle$$

where $P_{2}$ is the second Legendre polynomial, and *θ* is the angle between the director and the long axis of a molecule in the phase which is schematically shown in Figure 1. The brackets denote an ensemble average over many molecules. Figure 2 shows the impact of the order parameter on the polymer shape.

**Figure 3:**
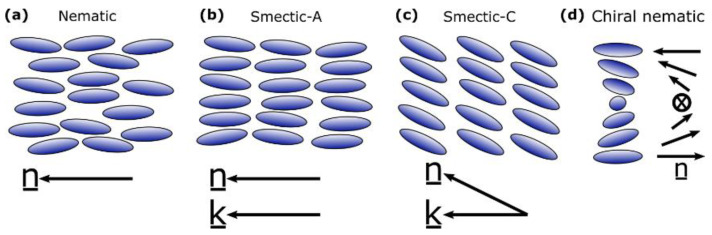
Schematics of (**a**) the nematic phase, (**b**) the smectic-A and (**c**) smectic-C phases in which the director, ($\underline{n}$), is parallel to and at specific angle to the layer normal (*k*), respectively, (**d**) the chiral nematic phase which has a characteristic pitch [8]. The black arrows indicate the director in each case.

Liquid crystal mesophases can be further characterised as different phases with different positional and orientational order. The nematic phase is a mesophase in which the molecules exhibit orientational but not positional order. The smectic phases additionally have translational order (a layered structure) with subtleties in the packing symmetry, allowing for many different variants; the simplest is the smectic-A phase in which the director coincides with the layer normal. Molecular chirality further modifies the symmetry of the system and can lead to distinct phases such as the chiral nematic phase, where the molecules adopt a helicoidal structure [7]. A schematic of these phases is displayed in Figure 3 [8]. 

There are three main material classes that combine the properties of polymers and liquid crystals, such as: conventional liquid crystal polymers, liquid crystal oligomers and LCEs [6]. LCEs are weakly crosslinked, so they have a low density of crosslinks between the polymer chains, resulting from the polymerisation of liquid crystalline monomers [1]. LCEs were first discovered by Finkelmann in 1981 [9], who reported LCEs exhibiting three distinct phases (chiral nematic, nematic and smectic). LCEs are thermotropic and typically lose their liquid crystal order at temperatures above the liquid crystal-isotropic phase transition (they are then referred to as isotropic liquid crystal elastomers). In some cases, the LCE can maintain the liquid crystal order up to very high temperatures—thermally degrading before an isotropic phase can form. Although LCEs display properties of both liquid crystals and elastomers, some of their properties are associated with neither category, such as semi-soft elasticity and spontaneous shape change [1,10,11].

The unique behaviour of LCEs arises from the coupling of the intrinsic liquid crystal orientational order to the distribution of the polymer backbone. In an isotropic system, the statistical average of the polymer backbone will be spherical and can be described by a single radius of gyration, R. When there is nematic ordering, the polymer backbone will form a spheroid which is elongated either parallel or perpendicular to the nematic director. In the former scenario, the polymer backbone distribution is said to be “prolate” and the radius of gyration parallel to the director, $R_{∥}$ is greater than the radius of gyration perpendicular to the director, $R_{⊥}$. In the later scenario, the polymer backbone is said to be “oblate” radius of gyration parallel to the director is smaller than the radius of gyration perpendicular to the director ($R_{∥}\;<\;R_{⊥})$. The polymer backbone shape is coupled to the order of the liquid crystal—so as the order is changed (for example from heating the LCE from the nematic phase), the backbone shape is changed. In the case of the nematic–isotropic phase transition, the backbone transforms from an anistropic oblate or prolate shape when in the nematic phase to an isotropic spherical shape when in the nematic shape—this is an example of actuation. This coupling of order to the backbone shape is responsible for the shape actuation properties of the LCEs. Reducing the intrinsic liquid crystal orientational order (as a result of changes in optical, chemical and thermal stimuli) reduces r (which is the ratio ($R_{∥}/R_{⊥}$)) to unity. This in turn results in the contraction of the LCE parallel to the director [1]. 

LCEs can be prepared in one of two geometries—polydomain or monodomain. Monodomain refers to an LCE where the director is uniform throughout the sample, as opposed to a polydomain LCE, where the director varies with position throughout the sample. The difference between the properties of monodomain and polydomain aligned LCEs include the ability for LCEs to display mechanical anisotropy (and hence the observation of semi-soft elasticity and/or the Mechanical Fréedericksz transition) and thermomechanical actuation. Additionally, light is strongly scattered at domain boundaries; therefore, polydomain LCEs tend to appear opaque, unlike monodomain LCEs. Without very special precautions during fabrication, liquid crystal elastomers are always found in polydomain form, with very fine texture of director orientations [12]. 

The intrinsic semi-soft elasticity (SSE) of liquid crystal elastomers is the second property which makes it ideal for use in biomedical applications. This is observed when tensile stresses are applied to an LCE perpendicular in direction to the LC director. Semi-soft elasticity corresponds to limited/no increase in the free energy of the system throughout a region of the deformation. This lack of increase in free energy corresponds to a plateau on the stress strain-curve of an LCE. Along the length of the plateau, a rotation of the nematic director supplements this deformation. This process offers a second route by which a 90° rotation of the director can be mechanically induced for a LCE under tensile strain. The Warner and Terentjev theory describing SSE also assumes a constant nematic order parameter throughout the deformation. An alternative deformation—the Mechanical Fréedericksz transition (MFT)—proposes that the director rotates sharply at a critical strain (as opposed to gradually over a strain range as is observed in materials displaying SSE behaviour) [1,13]. The Mechanical Fréedericksz transition is explored much less frequently in literature, so, although the relationship between the plateau in the stress-strain curves of LCEs and the director rotation in such materials is widely explored, this understanding is much more limited in LCEs displaying the MFT deformation [14,15].

The semi-soft elasticity, in turn, also results in LCEs behaving as ideal candidates for shock absorption applications, as a result of the internal relaxation of nematic director modes which provides an increased resistance to deformation. When considering the damping properties of LCEs, the mechanical response as a result of compressive loading, is key. In practice, as shown by Agrawal et al. [16], when a polydomain LCE is compressed dynamically, it can result in the stiffening of such materials as the director rotates in response to the dynamic load. This stiffening is a consequence of the coupling of the polymer backbone to the director resulting in a transformation from a prolate to an oblate backbone. The shock absorption properties of a material depend upon the tan(δ) of the material, where δ is the characteristic phase difference between the storage (elastic) modulus and loss (viscous) modulus [17]. When the elastic modulus is higher, tan(δ) increases and hence the shock absorption properties improve. An increase in stiffness increases the storage modulus, therefore increasingthe shock absorption properties. The shock absorption properties of monodomain and polydomain LCEs are different —when the compressive force applied to the LCE is parallel to the director alignment, the stiffening behaviour (similar to what was seen by Agrawal et al. [16]) can be observed. This director rotation is still observed in polydomain LCEs, but less director rotation is necessary to reach director alignment. However, when the compressive force is applied at a direction perpendicular to the director, this densification/stiffening/enhanced damping effect is not observed as there is no director rotation [18,19]. 

In 1975, de Gennes predicted the reversibility of the shape change responses of LCEs [20]. A reduction to the order parameter, via external stimuli, reduces the anisotropy of the backbone shape and, therefore, results in a reversible macroscopic shape change. These changes in order can result from the application of external stimuli, such as the application of heat, electric fields, or even light [21].

The behaviour and properties of the LCEs depend directly upon the final chemical structure of these elastomers and, therefore, upon the chemical synthesis routes required to produce the elastomers, as well as processing routes of these elastomers (i.e., techniques such as stretching and injection moulding to produce the desired elastomer shape). There are two main types of LCE: the first being where the side (pendant) groups are the mesogenic units, known as side-chain LCEs, and the second being LCEs where the mesogenic group is incorporated directly into the polymer backbone, known as main-chain elastomers [22,23]. 

Main-chain LCEs are traditionally synthesised by step-growth reactions, such as polycondensation and polyaddition reactions [24]. These reactions are traditionally difficult to control, and generally yield LCEs with high transition temperatures. Moreover, the materials produced are very polydisperse, meaning reproducibility often becomes an issue. However, recent developments in LCE synthetic chemistry have allowed these obstacles to be overcome, for example, the photo-crosslinking of functionalised main-chain polyesters [25], photopolymerisation of acrylates [10], cross-linking of functionalised liquid crystal polymers, [10] and hydrosilyation reactions [26]. More recently “click reactions” have been used for LCE synthesis [11,19,27,28]. “Click chemistry” is a term that was introduced by K. B. Sharpless in 2001 to describe reactions that are high yielding, wide in scope, stereospecific, simple to preform, create only by-products that can be removed without chromatography, and can be conducted in easily removable or benign solvents [29]. The Michael-addition of a nucleophile (e.g., thiol) to a α,β-unsaturated carbonyl compound (e.g., acrylate) has recently been studied in detail as a powerful “click” synthesis for LCEs. Many side-chain LCEs are generally siloxane-based, which are produced by hydrosilylation whereby a silyl hydride is inserted across an unsaturated bond [30,31,32].

There are extensive techniques that have been explored for LCE monodomain alignment. Some of these include: mechanical rubbing [28,33], magnetic [13,34], and electric fields [35,36], photoalignment [37,38], and stress alignment [39,40]. More recently, however, advanced alignment techniques have been explored. For example, exchangeable covalent bonds, which can undergo cleavage formation in the presence of external stimuli, such as heating or UV irradiation, have been introduced into LCEs to enable the programmability of the orientation of mesogens. The exchanging reaction of these covalent bonds can induce permanent rearrangement of polymer networks experiencing deformation, and removal of the stimuli quenches the reaction, fixing the alignment of the liquid crystal mesogens in the network [41]. Additionally, Jampani et al. [34], demonstrated alignment resulting from an osmotic pressure gradient whilst synthesising an LCE actuator shell. Impressively, they were able to achieve a negative order parameter as a result of this osmotic pressure gradient-based alignment route [42]. This followed the discovery of a negative order parameter in an acrylic-based nematic liquid crystal elastomer. When the strain direction was perpendicular to the nematic director and when the elastomer was strained enough, it resulted in a negative order state, coinciding with the auxetic behaviour of the LCE. This will be discussed in more detail later in this article.

## 3. Liquid Crystal Elastomers in Nature

In order to examine the significance of LCEs in biomimetics, the best place to start is to consider LCEs found in nature. While the discussion to date has mostly concerned thermotropic LCEs, most LCEs in nature are lyotropic, where the properties are tailored using the concentration of the two components rather than temperature. There is strong evidence that suggests that fibrils of transversely banded collagen can be regarded as LCEs [43]. There is extensive data which shows they are lyotropic in terms of the liquid crystalline structure, assembly and elastomeric properties. Woodhead-Galloway and Knight [44] studied elastoidin, a form of collagen isolated from shark fins, and they found that it has a high shrinkage temperature and high insolubility, indicating that the molecules are cross-linked. X-ray diffraction data of elastoidin and rat-tail tendon fibrils displayed sharp Bragg peaks and diffuse peaks, which was then confirmed to be indicative of lyotropic liquid crystalline behaviour by Hukins [45,46]. Many other natural fibrils have been shown to display liquid crystallinity, such as dogfish EC collagen [43], type 1 procollagen, [47] and other fibrillar collagens [43]. The mechanical behaviour does not on first glance corroborate LCE-like behaviour, because structures with collagen fibrils are composites and, therefore, their mechanical response depends upon the properties of the interfibrillar matrix. However, the isolated collagen fibrils themselves show an elastic response, and X-ray studies on the effect of strain of collagen fibrils have displayed reversible extensions of the axial period during cyclical loading in the “toe in” region of the stress–strain curve, as well as an increase in lateral molecular order accompanied by a reduction of mobility with strain [48]. The extension of the axial period is thought to result from the straightening out of thermally activated molecular kinks. This tensile response is consistent with what is observed by LCEs.

**Figure 4:**
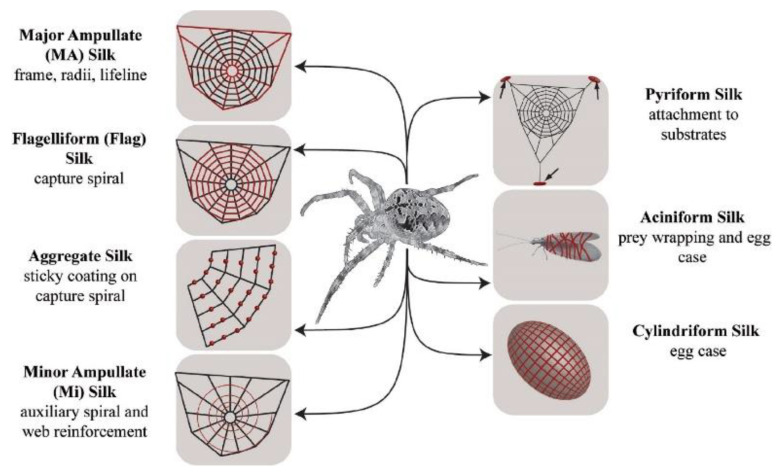
Schematic overview of different silk types produced by female orb-weaving spiders (Araneae). Each silk type (highlighted in red) is tailored for a specific purpose. Reproduced with permission from Elsevier from Eisoldt et al. [49].

**Figure 5:**
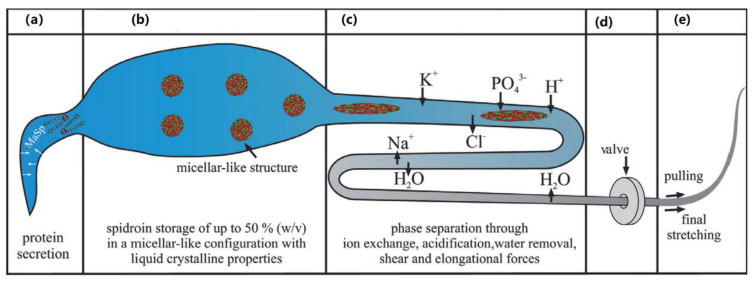
The spinning process that takes place in a spider’s spinning gland, reproduced with permission from Elsevier from Eisoldt et al. [49], where (**a**) is the tail, (**b**) is the ampulla (sac), (**c**) is the duct, (**d**) is the taper and (**e**) is the exterior.

There is also strong evidence to suggest spider silks have a LCE structure. A spider can produce a variety of silk materials. The protein which constitutes this silk is produced in major ampullate (MA) glands, and the resulting silk is often referred to as MA silk or “dragline silk”—this is illustrated in Figure 4 [49]. The capture spiral of an orb web comprises of fibres of only one type of protein which is produced in the flagelliform (Flag) gland of spiders and is hence known as flag silk. Spiders secrete a protein rich mixture (the proteins are referred to as spidroins, and the secretion is known as spinning dope), and the proteins have an unfolded structure when in the duct. The proteins assemble rapidly as they pass through a spinning duct, where phase separation occurs and then a mechanical drawing process occurs, wherein the rapid assembly of the silk commences and the laminar flow through the spinning duct promotes alignment of the proteins [50]. This process is illustrated in more detail in Figure 5. The alignment along the duct, combined with the high concentration, results in liquid crystal behaviour of the spinning dope [51], which in turn is used to form the LCEs.

Flag silk is highly elastic (it can be strained up to 300% before straining) and has a toughness of 150 MJ/m^3^, which is higher than carbon fibre and steel [52]. It also effectively dissipates the impact energy of prey. Dragline silk displays properties which are consistent with what would be expected of a lyotropic LCE. It displays a non-linear tensile behaviour [50] and X-ray data shows an axial period which increases reversibly when strained by 10% [53]. Dragline silk reveals a tensile strength that is comparable to Kevlar (4 × 109 N/m^2^) coupled with large elastic strain deformations when compared to Kevlar (35% of failure strain compared to 5%, respectively), indicating a short-range elastic response [49].

Spider silk is not suitable for commercial use as the amount of silk produced by a spider is very low and is only suitable for them to make their orbwebs – not for large scale industrial processes. Additionally, spiders are quite territorial and often end up killing each other, making it difficult to farm silk from them [54].

## 4. Biomedical Advances Inspired by LCEs

Both the spinning process used by spiders and the composition and structure of the silk they form can be considered when producing biomimetic materials with properties comparable to spider silk. The liquid crystal spinning of silks was modelled by using nematodynamics, nematostatics, and interfacial thermodynamics, and the resulting semi-quantitative prediction was consistent with the birefringence observed in the native spinning gland [55]. 3D printing is one of the more recent developments in LCE manufacturing, and it has a variety of advantages, including the ability to construct a wide range of geometries, as well as precise spatial deposition and temporal control [56]. In many ways, this technique shares similarities with silk-spinning. Silk fibres are spun into structures such as orbwebs and cocoons, in the same way 3D printing extrudes polymers/elastomers or other curable materials into 3D structures [57]. 

The exemplar qualities of silk, in theory, makes it an ideal biomimetic model material. The efficiency at which this silk is produced, under green conditions, makes the spinning process an attractive bionic model. Mimicking the chemistry and processing route would potentially allow a material with exceptional properties to be produced [58]. There has been long term interest in recreating this silk synthetically, yet, so far, no synthetically produced silk exhibits the exceptional properties of naturally spun silk. 

Spinning-inspired bionic methods include wet-spinning, dry-spinning and electrospinning [59]. Dry-spinning most closely resembles silk produced by spiders. Despite no technique existing to replicate dragline silk, methods to regenerate natural silk for uses in applications have been developed. Ling et al. [60] produced polymorphic regenerated silk fibres using a dry-spinning process; firstly, they developed a nematic silk microfibril solution which was both highly viscous and stable. They achieved this by partially dissolving silk fibres into microfibrils. This solution effectively behaves as the “dope”. It is then spun into regenerated silk fibres by direct extrusion in air. The modulus of the silk formed is even higher than that of the LCE formed by spiders [60]. Despite the impressive properties of the silk produced by the regeneration progress, it is limited by the requirement of input silk produced by spiders.

It is difficult to synthesise the silk produced by spiders for a range of reasons: the silk proteins created using genetic engineering and recombinant technologies have not been based on full-length spider silk gene sequences. Also, an incomplete understanding of the natural spinning processes, and the influences of the internal and external environment over silk properties, are limiting factors. In the regenerated silk process the main limiting factor is the spinning dope – using silk produced by spiders as an input to the process is unfeasible.The synthesis of the constituent proteins of natural spider dope would normally overcome this problem, but this is very difficult to achieve for a multitude of reasons. The proteins from the dope would normally be produced by recombinant protein expression, this involves injecting spider silk genes into bacteria, resulting in the bacteria expressing the proteins. This would be possible if the full-length sequences of spindroin-encoding genes were known for spiders but, unfortunately, at the present time, they are not. Attaining full-length spider silk proteins by recombinant expression is difficult because the silk proteins are large and consequently it is challenging for the bacteria to secrete them. Therefore, researchers have struggled to isolate and purify silk proteins using this method. There have been advances to try and overcome this, such as genetically modifying proteins secreted by bacteria, for example: *E. coli* [61], yeasts [62], plants such as tobacco [63], or in animals [64]. However, the proteins produced as a result of this vary in structure from native spidroins.

A recent advance using recombinant proteins has replicated the mechanical properties of dragline silk, using proteins containing 192 repeat motifs of the Nephila clavipes dragline spidroin. Although the spidroin varied noticeably in structure to what is naturally found in spider ducts, the similarities were significant enough to develop silk with the following properties: tensile strength (1.03 ± 0.11 GPa, where Dragline silk is 1.3 GPa), modulus (13.7 ± 3.0 GPa, where Dragline silk is 2.2 GPa), extensibility (18 ± 6%, but around 40% for Dragline silk), and toughness (114 ± 51 MJ/m^3^, but 180 MJ/m^3^ for Dragline silk) [61].

Researchers at Nexia Biotechnologies have successfully shown that transgenic goats can express the genes to spin spider silk, which is produced in their milk. This silk can subsequently be removed from the milk and purified [65].

## 5. The Significance of Liquid Crystal Elastomers for Understanding Biological Systems

An example of LCEs significance in biomimetics is their potential to lead towards artificial morphogenesis. Morphogenesis is a biological process that translates nanoscale details of molecular organisation into a macroscopic shape of an organism. For example, units of carbon, hydrogen and nitrogen assemble into molecular structures such as amino acids whose code ultimately makes up the structure of larger proteins, and it is these proteins that are ultimately the building blocks for eukaryotic and prokaryotic cells. Effectively, it is the process that causes cells, tissue and organisms to develop their shape. Morphogenesis is a mechanical process involving forces that generate mechanical stress, strain, and movement of cells, and can be induced by genetic programs according to the spatial patterning of cells within tissues. Therefore, ultimately, it is the process of creating complex 3D structures upon the exposure of external stimuli [66]. Scientists have been inspired by this and have attempted to mimic this process artificially. The classical approach to this is to take a 2D structure, which is folded or cut in certain directions, and transform it into a 3D structure, just like Japanese origami or kirigami. With isotropic materials—as in the case of origami or kirigami, which uses paper—this is relatively straightforward to maintain the intrinsic geometric properties. However, in the case of non-flat surfaces, it is not straightforward to upgrade them to 3D structures. Aharoni et al. [67] published an explicit protocol for pre-programming any desired 3D shape into a 2D LCE sheet [68], following a series of articles studying self-folding 3D LCE sheets [69,70,71]. Given an arbitrary 3D design, they demonstrated how to produce a flat sheet that can buckle into the desired shape when heated and return to flat when cooled—reversibly, due to the pre-designed molecular orientation on the film. A flat piece of paper cannot easily achieve a non-zero surface curvature which an LCE can. Patterning a director profile can create grooves and valleys in the surface of the LCE upon heating. When an LCE is heated, there is shrinkage along the director, $\underline{n}$, and therefore expansion in the perpendicular directions. So, if the patterned director field is circular, the perimeter contracts and extends, forcing the formation of a cone. The smooth variations in director field can successfully produce 3D shapes, such as spherical, pseudospherical, and toroidal surfaces. Ahrahoni demonstrated many examples of this—using a thiol-acylate-based synthesis route to produce LCEs that are flat at room temperature, but transform into the 3D shapes prescribed by the director distribution upon heating [67].

## 6. Liquid Crystal Elastomers for Biomedical Applications

**Figure 6:**
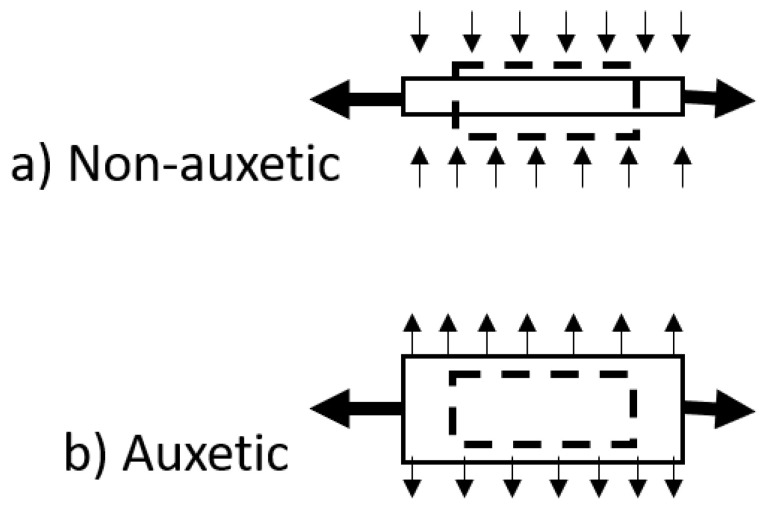
Schematic diagram of positive and negative Poisson’s ratio deformation. (**a**) Non-auxetic behaviour in which an initially undeformed material (dashed outline) undergoes longitudinal extension and lateral contraction (solid line) for a tensile load applied in the longitudinal (x) direction. (**b**) Auxetic behaviour in which an initially undeformed material (dashed outline) undergoes longitudinal and lateral extension (solid line) for a tensile load applied in the longitudinal (x) direction.

The Poisson’s ratio (*ν*) of a material is defined as the negative ratio of the transverse strain to the axial strain in the direction of loading. For many materials, this value is positive and reflects a need to conserve volume. Materials with a negative Poisson’s ratio display the unexpected property of lateral expansion when stretched, rather like a Hoberman^®^ sphere, with an equal and opposing densification when compressed [72,73]. Figure 6 presents a schematic of the behaviour of auxetic and non-auxetic materials. 

Materials with a negative Poisson’s ratio are referred to as auxetic materials. The vast majority of auxetic materials are cellular auxetics. Cellular auxetics are porous materials, where a volume increase results from the reorganisation of the internal cellular structure and a reduction in density. The auxeticity in molecular auxetics, however, arises from changes in their microstructure, perhaps resulting from molecular segments rotating upon deformation [74]. In molecular auxetics, only one axis shows a negative Poisson’s ratio, and this is coupled to a large contraction in another axis to maintain constant volume and the original sample density.

Until recently, molecular auxetics were only found in nature, for example, in 69% of cubic metals, α-cristobalite, and numerous examples of the zeolite class of materials [75,76,77]. Cellular auxetics are abundant in nature; for example, this behaviour has been reported in cow teat skin [78], cat skin [79], cancellous bone [80], tendons [81] and membranes found in the cytoskeleton of red blood cells [81,82]. Unlike molecular auxetics, many examples of synthetic cellular auxetics exist, such as the Hoberman^®^ sphere [72], ultra-high molecular weight polyethylene (UHMWPE) [83] and paper [84].

Recently, studies have identified the benefits of using auxetic materials in skin healing [85]. Skin healing is facilitated by the migration of cells to the wound site. Small simple wounds, such as small finger-pricks, rarely need assistance in healing. However, when complex wounds occur, such as burns, or when more widespread tissue regeneration is required, assistance can promote faster healing and minimise scarring. Tissue regeneration can be facilitated if a suitable scaffold is provided, the desired cells replicate more easily and grow along the predefined structure. Polylactic acid-based fibres which can be produced by electrospinning are cellular auxetics [85,86]. The auxeticity of these scaffolds are particularly attractive because of their ability to apply an enhanced negative pressure on the wound site. 

Auxetic skin sensors have also recently been developed [87]. The auxetic nature of these skin sensors is particularly appealing because they display excellent mechanical compliance to dynamic body motions. Human skin displays a negative Poisson’s ratio in some regions—expanding biaxially during bending, exhalation and muscle tension. Often, polymer films are unable to maintain contact with the skin under large body motions due to their positive Poisson’s ratio. The usage of auxetic sensors can therefore maintain conformational contact [87].

**Figure 7:**
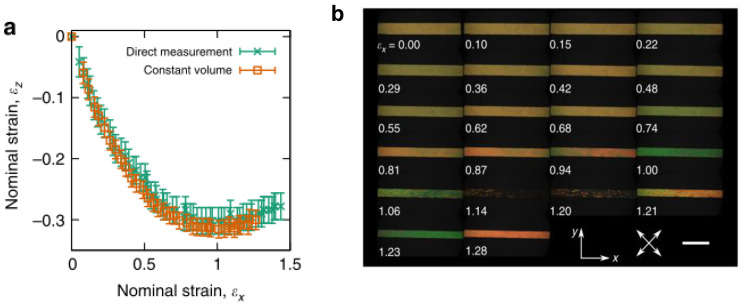

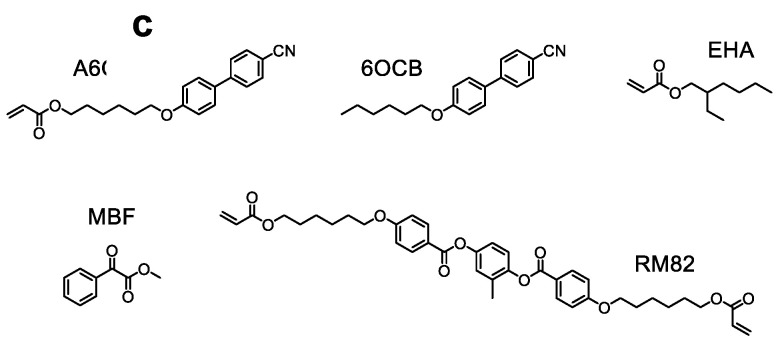
(**a**) z-axis strain, ε_z_ in response to imposed x-axis strain, ε_x_, measured via direct observation of the polyacrylate-based auxetic LCE in the xz plane (green crosses) and through the application of the constant volume condition to strain measurements of the xy plane (orange boxes). Errors are measurement errors (*n* = 1). (**b**) Negative order from polarising microscopy. Polarising microscopy textures at different strains. The birefringence colours indicate the retardance initially decreases, becoming zero at ε_x_ = 1.14, before increasing again. Scale bar, 5 mm. (**c**) shows the constituents used to synthesise the auxetic LCEs reproduced with permission from Nature from Mistry et al. [2].

Recently, a side-chain LCE was the first ever synthetic material to display molecular auxetic behaviour [2]. This polyacrylate-based LCE showed that volume was conserved when strained, confirming that the material was a molecular auxetic. The Poisson’s ratio displayed by the material was up to −0.8. The auxetic behaviour was not observed at low strains, but at higher strains (>80%). Once the strains necessary to display auxetic behaviour of the LCE were reached, a coincident negative order parameter was also observed. It is believed that the auxetic response is related to the inherent Mechanical Fréedericksz transition displayed by some LCEs [2]. Figure 7 shows the change in the thickness (strain in the z-direction, ε_z_) of the polyacrylate based auxetic LCE in response to strain in the x-direction, ε_x_. Shown also is the change in birefringence as a function of strain, displaying a decrease in retardance as the strain is increased, reaching a minimum at ε_x_ = 1.14, where what is believed to be a negative order parameter is observed. A further increase in ε_x_ results in the retardance increasing again. The relationship between the liquid crystal order and the emergence of auxetic behaviour of the LCE is still being explored. The reason as to why some LCEs display the Mechanical Fréedericksz transition whereas others do not is also currently unclear.

These auxetic LCEs show exciting potential for biomimetic applications. As previously discussed, the auxeticity of materials used in skin sensors and skin healing scaffolds significantly enhances their performance compared to the non-auxetic counterparts. Molecular auxetics could be used in composites which mimic the stress-strain and auxetic behaviour of human skin [87]. Conventional auxetics have an “open” microstructure, which confers reduced mechanical properties [77]. In many cases, these “open” microstructures display a tensile strength that is too weak for practical applications due to their microscale porosity; however, these limitations could be circumvented by molecular auxetics due to their “closed” microstructure.

Another potential application is the use of LCEs as artificial blood vessels; the auxeticity would allow the vessels to withstand the high pressure of blood through the vessels, which are less prone to rupture as a result of thinning [88]. 

Furthermore, a range of tissues display auxetic behaviour [81,82]. The discovery of the inherent auxeticity of these tissues has a range of implications for tissue engineers in mimicking the properties of these auxetic tissues. The mechanical characteristics of engineered tissue ideally should match or enhance the mechanical properties of healthy, normal host tissues, permitting full functionality, enabling it to fulfil its role in vivo. Cells exist in their natural in vivo environment embedded within an extracellular matrix, which is the natural scaffold of the body produced by the cells within tissues. Therefore, if the target tissue is auxetic, an auxetic scaffold would most closely match the properties of this tissue. The matching of this characteristic would be beneficial in recreating the loading environment that cells would naturally experience. As will be discussed in further detail later in this review, LCEs have already been considered and have shown to be suitable for use as tissue replacements, as their actuation properties make them ideal candidates for use as artificial muscles. Exploiting the auxeticity of some LCEs is, therefore, promising for potential applications in tissue engineering.

This discovery of a molecular auxetic has overcome a long-standing limitation in the auxetics industry and there is, therefore, a lot of potential for these materials not just in biomedicine, but in the wider materials world [89,90]. The main challenge lies within understanding and, if necessary, making adaptations to ensure the biocompatibility of the materials.

## 7. Liquid Crystal Elastomers in Tissue Engineering

**Figure 8:**
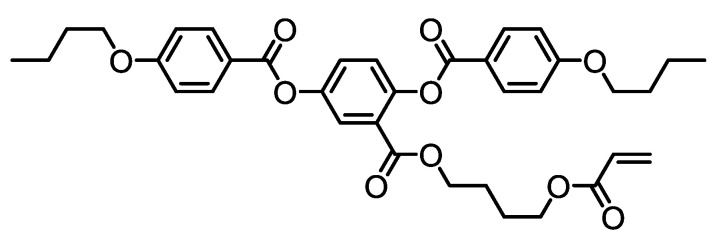
4′-acryloyloxybutyl 2,5-(4′-butyloxybenzoyloxy)benzoate), a polymerisable azobenzene-based compound used in various studies of photoresponsive liquid crystal elastomers [93].

The flexibility stemming from the elasticity of LCE polymer networks allow for a large and reversible anisotropic dimensional change in response to applied stimuli. Based on this, de Gennes proposed a theoretical study suggesting the possibility of using LCEs as artificial muscles [20]. He predicted that when the temperature is lowered below the nematic clearing point, strong uniaxial deformations occur. The estimated shape relaxation time is well within actual muscle contraction times. Küpfer and Finkelmann [91] then went on to experimentally confirm this behaviour, synthesising elastomers where all mesogens were oriented uniformly across the sample and, as a result of coupling, the polymer backbone was elongated along the director, creating a monodomain sample. The individual polymer chain shape changes were translated to a macroscopic shape change of the elastomer sample at the nematic–isotropic transition [92]. Following this, a range of studies have shown physical properties suitable for use in artificial muscles, and uniaxial deformations of up to 600% have been achieved. The main focus when considering the design of artificial muscles involves attempting to mimic the following properties displayed by muscles: (1) achieving a uniaxial contraction of at least 25%, (2) a stress exerted of at least 350 kPa, and (3) a contraction frequency of 5 to 10 Hz. Although many actuating LCEs can contract by up to 600%, it is rare for LCEs to fulfil all three properties [33]. Thomsen et al. [33] synthesised two LCEs using mesogens with laterally-affixed polymerisable side chains based on 4′-acryloyloxybutyl 2,5-(4′-butyloxybenzoyloxy)benzoate), the structure of which is shown in Figure 8. The elastomers exhibited behaviour that satisfied all three criteria necessary for artificial muscles: the relaxation frequency was between 5 and 10 Hz, they displayed strains between 35% and 45%, and finally they exerted a stress of 210 kPa, which is slightly lower than the average exerted by biological muscles. LCEs produced by reacting rigid-rod mesogenic epoxy monomers with aliphatic diacids of variable length displayed exceptional properties, with the exerted stress reaching an exceptional value of 12 MPa—by far exceeding the properties necessary for artificial muscles, strains of 310% and relaxation time of about 150 ms, which corresponds to a relaxation frequency 6.67 Hz [93]. Following this study, a wide range of materials have shown properties which display physical properties suitable for muscle replacement, with facile synthesis routes.

**Figure 9:**
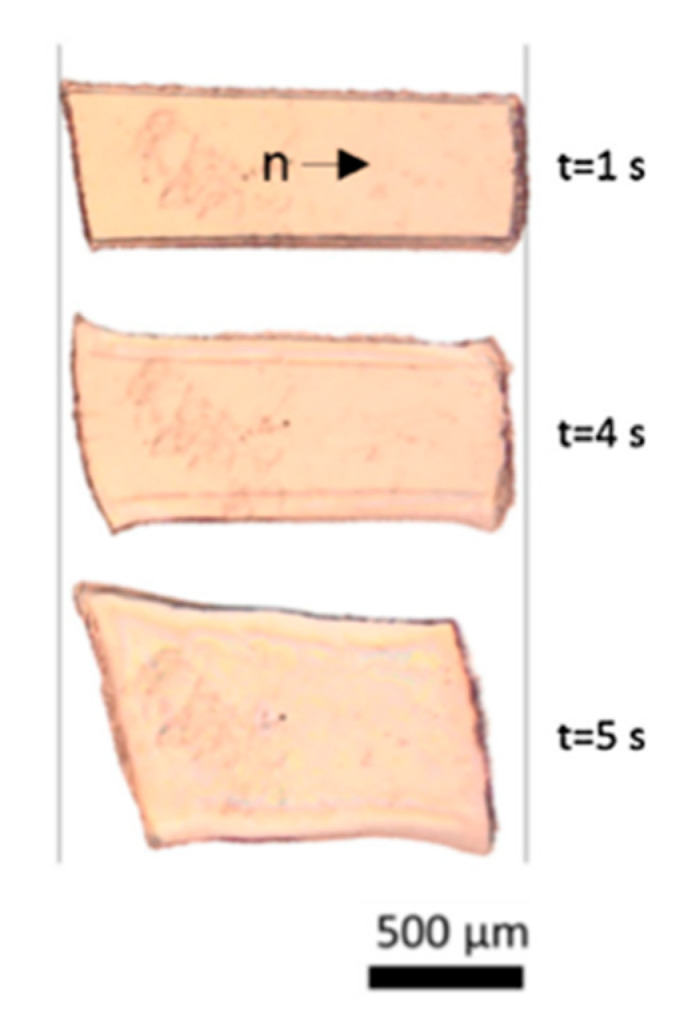
Images taken on an optical microscope show samples with planar alignment, 1, 4 and 5 s following immersion in tetrahydrofuran (THF). Reproduced with permission from Elsevier from Boothby et al. [94].

More recently, LCEs that are responsive to other stimuli, e.g., specific chemicals, have been developed, which also express the necessary properties for use as artificial muscles. Boothby et al. [94] developed an LCE which was not only temperature responsive, but also responsive to chemical stimuli. It displayed a contraction of 26.1% in response to dimethylformamide (DMF). It also displayed this behaviour with different contractions in other solvents such as THF, as shown in Figure 9. A limited amount of mechanical characterisation of this elastomer was reported, so we cannot accurately determine whether it is a suitable material to act as an artificial muscle. Nevertheless, the study is a good starting point for the development of chemoresponsive artificial muscles in the future [95].

Light-responsive LCEs with actuating properties have also been developed. These LCEs generally incorporate azobenzene groups. The azobenzene group exhibits both cis and trans isomers. When exposed to UV of a suitable wavelength, the trans azobenzene is converted into the cis conformer. The trans isomer can be considered rod-like, whereas the cis isomer has a bent structure. The rod-shaped molecules act to stabilise the liquid-crystalline phase, whereas the bent shape acts as a non-mesogenic impurity—so UV radiation can effectively initiate an isothermal phase transition from nematic to isotropic when incorporated in a liquid crystal. In the case of LCEs, UV irradiation can initiate expansion by up to 50% [92]. Historically, there have been multiple problems associated with the use of photo-responsive LCEs for actuation in artificial muscles—the relaxation time for the developed LCEs was generally far too long, often requiring many minutes for cis–trans conversion and hence relaxation back to the original shape. Recent developments have overcome this relaxation time limitation.

Recently, more focused applications of LCEs for use within the human body as tissue replacements have been studied. The use of light-assisted LCEs to assist cardiac contraction has recently been explored by Ferrantini et al. [96]. They firstly analysed the biocompatibility of these LCE-based molecules. LCEs are a very suitable candidate for such an application due to their impressive photo-mechanical actuation properties and the tunability of the tensile properties. The acrylate-based LCE was used as a culture substrate, and they successfully observed cell differentiation and maturation of different human and murine cell lines. They observed that human dermal fibroblasts and immortalised mouse myoblast muscle cell lines adhered successfully onto the films, and cell differentiation and maturation was observed. The muscle cell lines differentiated in long muscle fibres showing several nuclei and well organised actin fibres [86]. The findings of this biocompatibility study allowed for a more developed in situ study, determining the suitability of light-responsive LCEs to assist cardiac contraction [27]. In this case, the LCE was modified slightly to incorporate an azobenzene-based dye, the same dye discussed earlier with the structure shown in Figure 8. Unlike earlier studies on photoresponsive LCEs, even those incorporating the same dye, the response time of this azobenzene-incorporated LCE was sub-milliseconds, as it has a short half-time in the cis-state. This in vivo study involved the fabrication of these LCE films into thin strips, with dimensions comparable to those of thin cardiac trabeculae. They studied the mechanical behaviour of LCE strips by measuring passive tension and active tension in the air on stimulation with the green LED light source at its maximal intensity. The tensile response as a result of UV illumination was characterised, and under wide illumination, a strong tensile response, with no discernible decay, remained over 24 days, where the LCE was subject to flashes of UV radiation every second as an attempt to mimic cardiac twitches. To demonstrate the ability of the LCE in assisting cardiac contraction, the tensile response of a strip of mouse right ventricle trabecula was compared to a strip of mouse right ventricle trabecula mounted in parallel to an LCE strip (which herein will be referred to as the muscle/LCE sample). When the UV light was switched off, the tensile force exerted by the muscle/LCE sample was almost the same as the force exerted by the muscle alone. However, when the muscle/LCE sample was exposed to UV light, a force three times higher was recorded. This remarkable result demonstrates the ability for this LCE to provide significant systolic assistance. Additionally, the contraction time of the LCE/muscle sample was comparable to the contraction time of the muscle alone, and the 4 stages of the in vitro contraction process could be reproduced. A limitation of photoresponsive materials is that they are often subject to photo-degradation as a result of UV exposure, limiting their lifetime [97]. This example has focused on assisting cardiac contraction; however, changing the molecular parameters and the actuation stimuli may also allow matching of the features of skeletal or smooth muscles, extending this technology to non-cardiac applications. This is an in vitro demonstration of the suitability for LCE in biomimetics—the next step will be implementing such materials in vivo [96].

**Figure 10:**
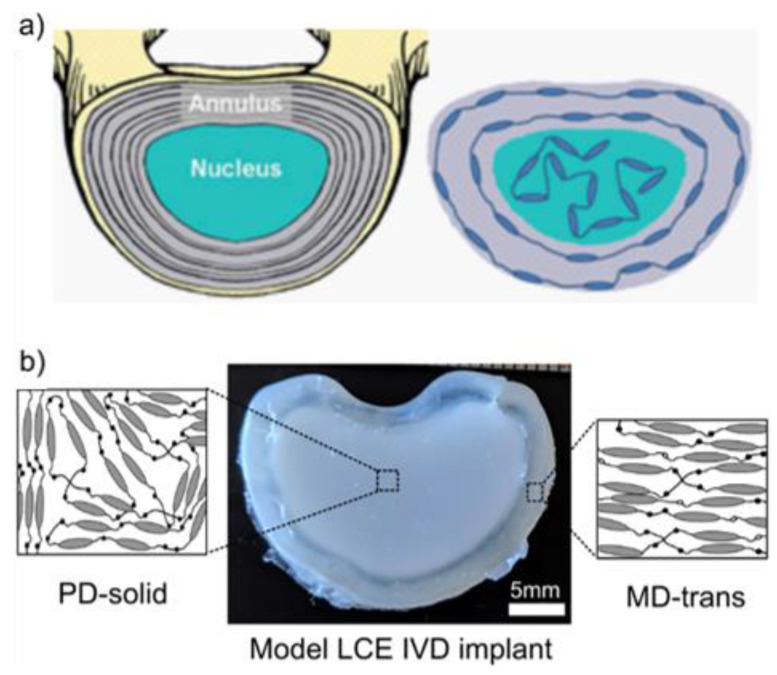
(**a**) A schematic diagram that shows the model intervertebral disc (IVD) was devised by mimicking the collagen alignment with the LCE’s mesogen alignment. (**b**) The model IVD implant made from LCE materials. Solid polydomain LCE was selected as an analogue for the nucleus pulposus, while transversely aligned monodomain material was selected as an analogue for the annulus fibrosus. The outer transversely aligned monodomain surrounded the central polydomain region. Reproduced with permission from Elsevier from Shaha et al. [19].

We have discussed the significance of LCE for tissue engineering focusing more specifically on their actuation properties for their use as artificial muscles. However, their exceptional properties do not limit them to use in muscles. For example, Shaha et al. [19] synthesised a replacement intervertebral disc based on LCEs. This application exploits both the load-bearing and impressive energy dissipation properties of LCEs. A polydomain LCE was used to mimic the nucleus pulposus and a transversely loaded monodomain liquid crystal elastomer (i.e., the direction of the compressive force was perpendicular the compressive force direction) was used to mimic the annulus fibrosus (see Figure 10 for a schematic of the intervertebral disc). The tensile and compressive tests of the transversely loaded monodomain LCE did not display semi-soft elastic behaviour as there was no bulk realignment of the director, so the material recovered rapidly when the applied load was removed. Therefore, it could provide support and structure for the central nucleus pulposus. A polydomain LCE was used to replace the nucleus pulposus as the semi-soft elastic behaviour meant it displayed exceptional damping properties as a result of director rotation. The damping and shear in terms of both the trend and magnitude of the polydomain LCE were very similar to human nucleus of intervertebral discs.

Materials with poor energy dissipation properties are unsuitable for use as replacement vertebral disks, as their use can lead to loosening and premature device failure due to wear and damage [19]. Shaha et al. utilised acrylate-based LCEs synthesised via a facile 2-stage thiol-acrylate click reaction which have previously demonstrated cytocompatibility. This in vivo study analysed a polydomain porous elastomer synthesised using a salt leaching technique, by adding salt crystals to the LCE forming mixture, as well as nonporous monodomain and polydomain LCEs. The biocompatibility of the LCEs was evaluated by implanting samples of the polydomain and monodomain LCE into a rat, and no noticeable swelling or inflammation was observed, indicating that the LCEs they synthesised were biocompatible. They demonstrated a proof-of-concept of the ability of a monodomain-polydomain LCE composite to mimic a vertebral disc. The centre of the implant comprised of polydomain LCE which displayed mechanical properties, such as the change in tan(δ) with frequency, similar to the nucleus of a vertebral disc. The outer ring of the disc comprised of monodomain liquid crystal elastomer, which displayed mechanical properties comparable to the behaviour displayed by the vertebral disc annulus (as seen in Figure 10). The in vitro testing of this device is still ongoing.

The in vivo testing of LCEs as tissue replacements has only just begun. We have observed how the concept of using LCEs as a muscle replacement has developed over the years, simply from being a theory to developing LCEs that display properties that appear to be suitable for tissue replacements. More recently, the usage of LCEs has shown success in vivo as muscle support devices, and in vitro tests for LCEs are still ongoing. However, this is just the beginning of making de Gennes’ vision of using LCEs a reality. Now that the multiple technologies have been developed, research will likely focus on making this a reality, with clinical trials testing their efficacy and, in time, we will perhaps observe LCEs used in humans as actual artificial muscle, or other tissue replacements.

Finally, LCEs have been studied extensively as promising candidates for cell scaffolds—materials that have been engineered to cause desirable cellular interactions to contribute to the formation of new functional tissues for medical purposes. LCEs can be synthesized to employ a porous architecture—this is vital to allow nutrient transport between cells. Synthesis techniques to produce porous architectures include microemulsion photopolymerization [98], salt-leaching [99] and electrospinning [100]. Additionally, a cell scaffold should allow mechanical control of cell behaviour. LCEs can achieve this due to the liquid crystalline ordering. Multiple studies have confirmed that LCEs can achieve cell alignment due to the inherent liquid crystalline order of LCEs—this is necessary for controlled and directional growth of cells [101,102]. The use of LCEs as cell scaffolds is discussed in great detail in a recent review by Prévôt et al. [3].

## 8. Conclusions

In 1975, six years before the synthesis of the first ever LCEs, the significance of LCEs was highlighted by de Gennes, who presented a theoretical framework of artificial muscle-base on the contraction of LCEs at the nematic-isotropic transition. Since then, incredible advances have been made in the field of LCEs to make this a reality. Studies have explored the possibility of stimuli-responsive LCEs, and in more recent years, in vitro and even in vivo testing has begun to take place, providing proofs of concept in the usage of LCEs as actual artificial muscles. Although they have not yet been used in a medical environment, the impressive developments thus far imply that this could be a possibility in the near future. The significance of LCEs in biology is not only limited to artificial muscles; a more recent discovery of a molecular auxetic LCE has also opened up the possibility for the use of LCEs as general artificial tissues. In addition to this, LCEs have, unsurprisingly, been found to exist in nature, providing exceptional physical properties. This highlights the need for further research on understanding the structure and behaviour of these natural LCEs, the findings of which may allow for the synthesis of materials with exceptional mechanical and tensile properties, potentially on a commercial level.
